# Fractured sternal wire causing a cardiac laceration

**DOI:** 10.1186/s13019-023-02452-6

**Published:** 2023-12-09

**Authors:** Matthew S. Khouzam, Kristina Jacobsen, Joseph H. Boyer, Ahmad Zeeshan, David Spurlock, Tomer Z. Karas, Jorge E. Suarez-Cavelier, Daniel Rinewalt, Linda Bogar, Scott Silvestry, George J. Palmer, Kevin D. Accola, Nayer Khouzam

**Affiliations:** 1 Loyola University Medical Center, Stritch School of Medicine, Maywood, IL USA; 2 Division of Cardiothoracic Surgery, AdventHealth, Orlando, Florida USA

**Keywords:** Hemopericardium, Broken sternal wire, Unstable sternum, Cardiac laceration, Case-report

## Abstract

**Background:**

Hemopericardium is a serious complication that can occur after cardiac surgery. While most post-operative causes are due to inflammation and bleeding, patients with broken sternal wires and an unstable sternum may develop hemopericardium from penetrating trauma.

**Case presentation:**

We present the case of a 62-year-old male who underwent triple coronary bypass surgery and presented five months later with sudden anterior chest wall pain. Chest computed tomography revealed hemopericardium with an associated broken sternal wire that had penetrated into the pericardial space. The patient underwent a redo-sternotomy which revealed a 3.5 cm bleeding, jagged right ventricular laceration that correlated to the imaging findings of a fractured sternal wire projecting in the pericardial space. The laceration was repaired using interrupted 4 − 0 polypropylene sutures in horizontal mattress fashion between strips of bovine pericardium. The patient’s recovery was uneventful and he was discharged on post-operative day four without complications.

**Conclusion:**

Patients with broken sternal wires and an unstable sternum require careful evaluation and management as these may have potentially life-threatening complications if left untreated.

**Supplementary Information:**

The online version contains supplementary material available at 10.1186/s13019-023-02452-6.

## Introduction

Hemopericardium is a potential life-threatening complication that can occur after a sternotomy [1]. Most cases of post-operative hemopericardium are caused by infection or bleeding within the pericardial space, leading to the accumulation of blood [2]. Penetrating and blunt trauma are also a widely recognized cause of hemopericardium, though are not often thought of in the context of post-cardiac surgery complications. We present a case of a 62-year-old male who underwent triple coronary bypass surgery and presented five months later with hemopericardium as a result of a broken sternal wire penetrating into the pericardial space causing a cardiac laceration.

## Case presentation

The patient is a 62-year-old Caucasian male with a history of surgical myocardial revascularization performed five months ago at a different institution who presented to our emergency department with a sudden onset of anterior chest wall discomfort radiating to his neck and both shoulders. The pain was associated with dizziness, nausea and diaphoresis. There were no precipitating factors. The patient took three aspirins without relief of his symptoms. His past medical history was significant for essential hypertension, dyslipidemia, non-insulin-dependent diabetes mellitus, chronic kidney disease, and gout. He had a previous left sided thoracentesis performed six weeks ago for a symptomatic left pleural effusion.

**Fig. 1:**
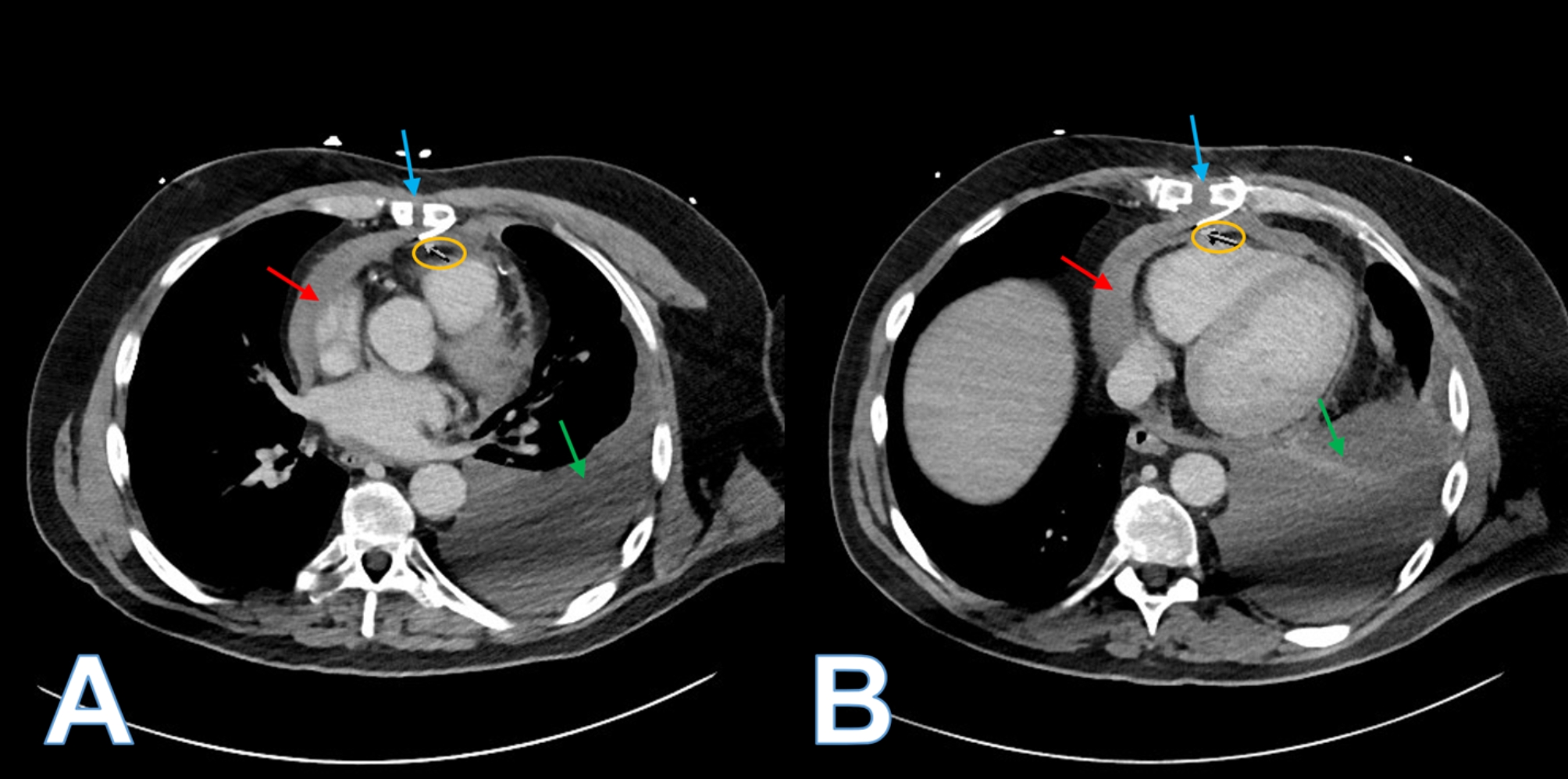
**A)** Computed tomography angiography at the level of the left atrium demonstrating sternal bone diastasis (blue arrow), fractured sternal wire angulating into the pericardial space (yellow circled arrow), hemopericardium (red arrow), and left pleural effusion (green arrow) **B)** Computed tomography angiography at the level of the diaphragm demonstrating sternal bone diastasis (blue arrow), fractured sternal wire angulating into the pericardial space (yellow circled arrow), hemopericardium (red arrow), and left pleural effusion (green arrow)

**Fig. 2:**
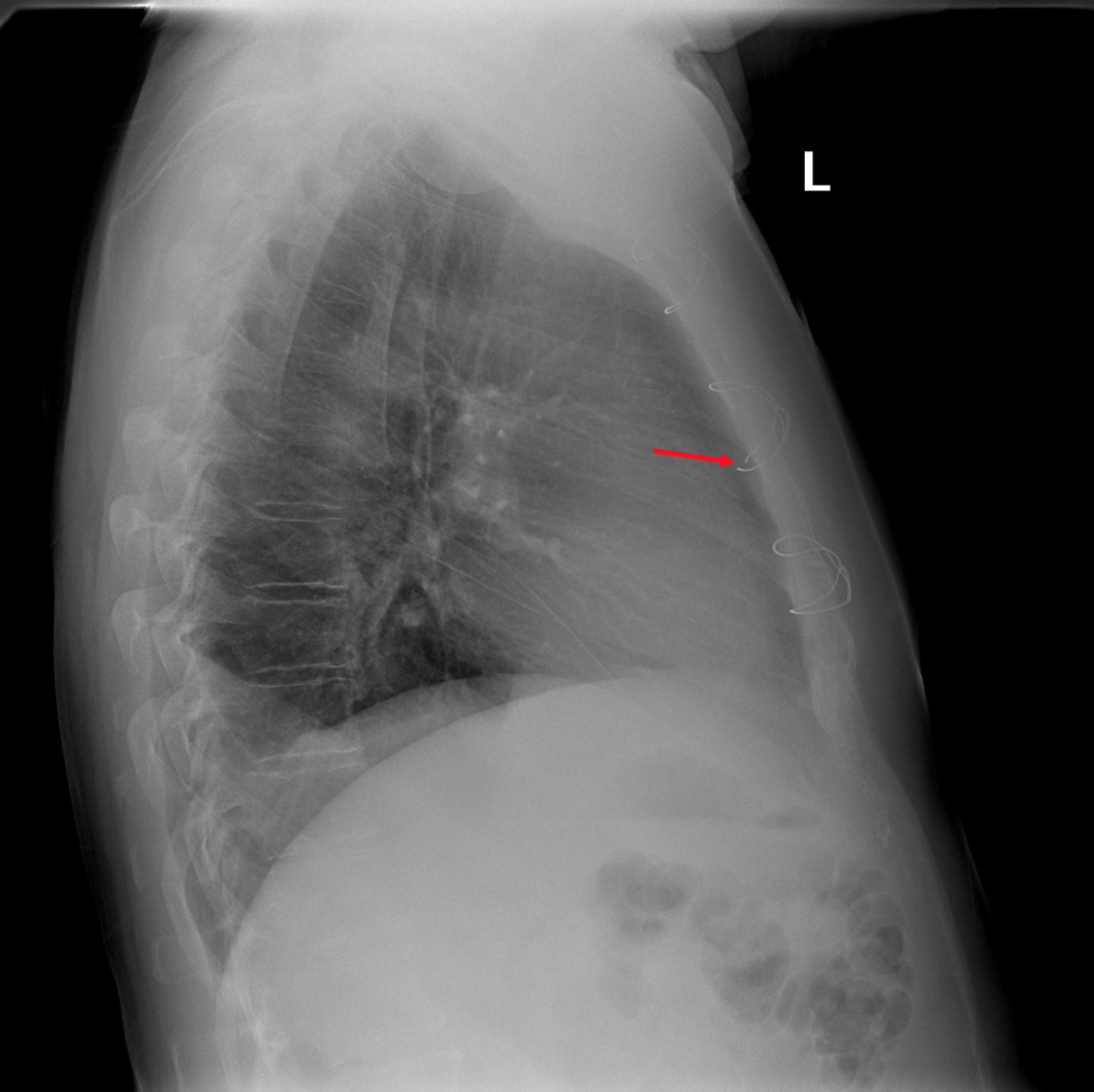
Lateral chest X-ray from five months prior demonstrating a fractured sternal wire located at the posterior sternal table (arrow)

On examination, the patient appeared acutely ill. He had a body mass index of 36.6 kg/m². He was diaphoretic. He was tachycardic with a pulse rate of 102 bpm, regular sinus rhythm on telemetry. His systolic blood pressure was 132 mmHg without pulsus paradoxus. He had no jugulovenous distention. There was visible paradoxical movement of his sternum that, according to the patient, has been present several weeks after his original surgery. An electrocardiogram showed no evidence of ischemia or acute injury. Chest roentgenograms revealed a large left sided pleural effusion and two fractured lower sternal wires (Fig. 1). It is also noteworthy that a chest X-ray from five months prior only revealed one broken sternal wire (Fig. 2). Echocardiography revealed a small to moderate sized hemopericardium, without evidence of tamponade physiology.

Computed tomography angiography (CTA) of the chest confirmed the large left sided pleural effusion with associated left lung atelectasis. There were also findings suggestive of sternal dehiscence with diastasis of the sternal bone. Two lower sternal wires demonstrated wire fragments which projected posteriorly and appeared to penetrate the anterior pericardial space. There was a small to moderate sized hemopericardium present. No active extravasation of contrast was present.

The patient was emergently taken to the operative suite. Intraoperative, transesophageal echocardiogram (TEE) confirmed a moderate-sized hemopericardium with blood and blood clots in the pericardial space. The clot was visualized overlying the right ventricle. There was no evidence of early, diastolic collapse of either the right atrium or right ventricle. Both right and left ventricular function were preserved. Shortly thereafter, his hemodynamics began to deteriorate, necessitating volume administration, and pressor support with epinephrine. Systemic heparinization, 4 mg/kg of heparin, was administered, and the patient was placed on femoral artery – femoral vein cardiopulmonary bypass.

**Fig. 3:**
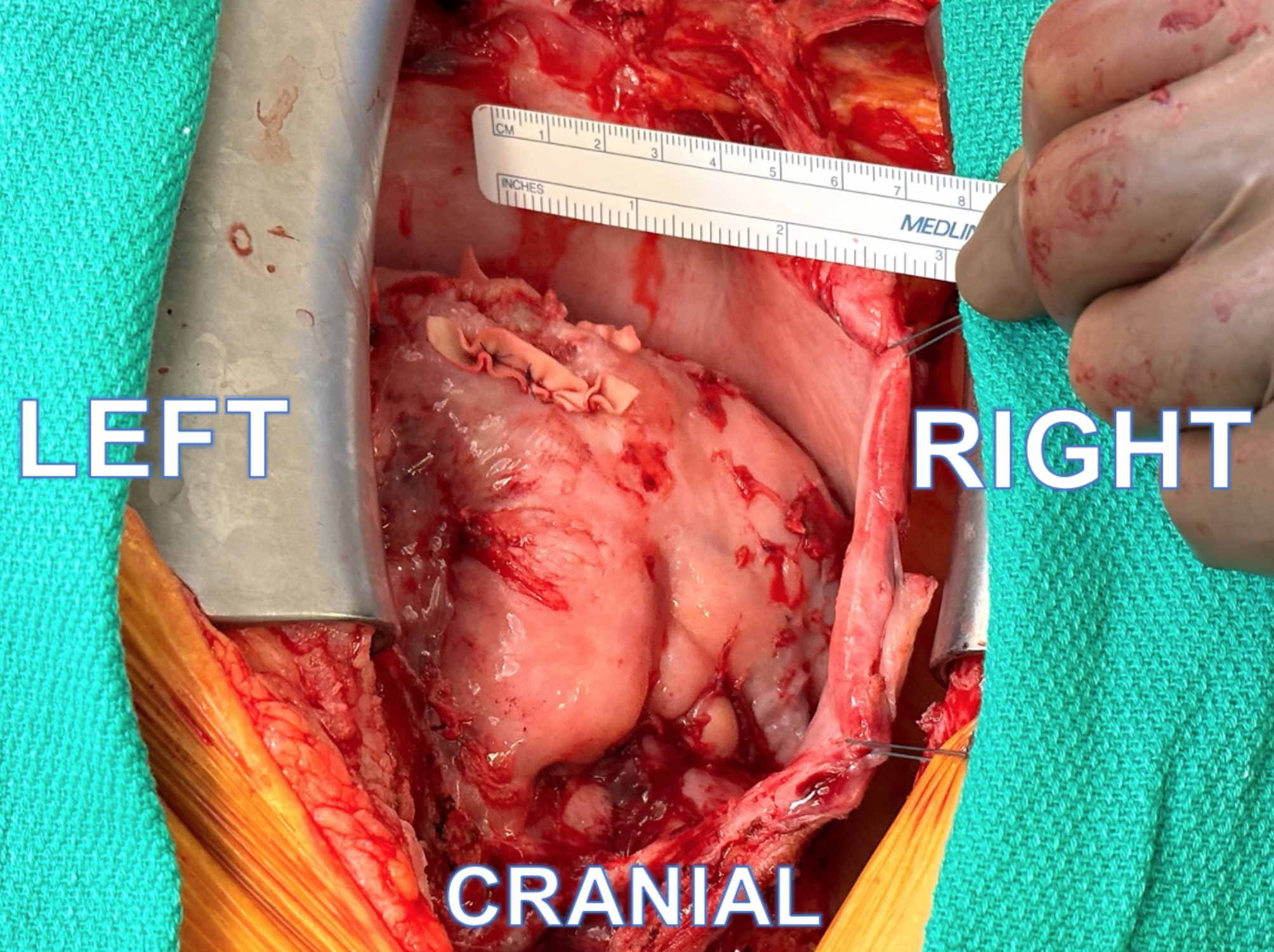
Surgical image demonstrating repaired jagged right ventricular laceration

A redo-sternotomy incision was made. All sternal wires and wire fragments were removed. Findings revealed complete dehiscence of the sternal bone from the manubrium to the xiphoid with a tremendous amount of granulation tissue present. Dark blood was welling up between the sternal bone halves. Dense mediastinal adhesions were present and lysed as encountered. Mediastinal exploration revealed a large clot overlying the free wall of the right ventricle. Beneath the clot, we identified a 3.5 cm bleeding, jagged right ventricular laceration that correlated with the CTA findings of the lower fractured sternal wire projecting in the pericardial space. Digital control of the laceration was performed. Limited dissection of the right heart was performed to allow a tension-free repair of the cardiac laceration. The jagged, right ventricular laceration was repaired using interrupted 4 − 0 polypropylene sutures in horizontal mattress fashion between strips of bovine pericardium (Fig. 3). Further inspection revealed no other pathology. After volume loading the heart and confirming that the repair was solid, the patient was easily weaned from cardiopulmonary bypass without difficulty. TEE revealed good biventricular function. The sternum bone was debrided and closed using sternal plates. The patient’s hospital course was uneventful and he was discharged on post-operative day four.

## Discussion

This case demonstrates a patient who presented with chest discomfort post-bypass surgery and was found to have a small to moderate-sized hemopericardium as a result of a fractured sternotomy wire projecting posteriorly into the pericardial space resulting in a cardiac wound. In our patient, the fractured and posteriorly angulated sternal wire is thought to have been a result of his sternal instability. This case highlights the importance of evaluating patients with an unstable sternum who have recently undergone a sternotomy as this can lead to a sternal wire fracture and potential angulation. Although sternal wire fractures occasionally appear on post-operative follow-up chest roentgenograms, they are often thought of as nonspecific findings or even omitted from the final radiology report [3–5]. Findings of central sternal lucency and loss of sternal wire alignment raises the suspicion for sternal nonunion/dehiscence. Moreover, this radiologic findings, particularly in the context of an unstable sternum, may result in the fractured wire angulating and penetrating the pericardium causing serious cardiovascular injury.

Post-sternotomy hardware complications include sternal wire rotation, migration, displacement, rupture, and fracture [6]. The incidence of each of these complications has not been elucidated. The mechanism for wire-fracture is hypothesized to be multifactorial, with mechanical distortion during sternal closure and chemical erosion from contact with bodily fluids being the main contributing factors [3]. The breaking of sternal wires has the potential to cause serious complications, with the literature reporting cases of fractured fragmented wire embolizing to the lung [7] and migration and puncture of the wire fragments into the ascending aorta [8]. We present however, to the best of our knowledge, the first reported case of a direct sternal wire puncture into the pericardial space.

Other, more common sequelae of post-sternotomy hardware complications include sternal instability and sternal dehiscence, which when combined, have an estimated prevalence of 1–3% [9]. There are several patient-specific risk factors that can contribute to sternal dehiscence, including chronic obstructive pulmonary disease, obesity, trauma, and diabetes mellitus. In addition, certain operative and post-operative factors may also increase the risk of sternal dehiscence, such as prolonged time on pump, procedures involving the internal thoracic artery, repeat surgery, and prolonged post-procedure ventilation. [10]. Sternal dehiscence is usually evident clinically, but may be clinically occult in a small subset of patients [4]. In the modern day, surgeons often close sternotomies by placing wires in figure-of-8 fashion, as it increases the area of contact between the wire and the sternum and is associated with lower rates of wire loosening or fracturing [11]. In high risk patients, some surgeons augment their sternal closures with additional hardware such as plates or clips, though recent studies have shown that these often have little to no impact on post-operative morbidity [12, 13].

Treatment of sternal wire fractures, particularly in patients with an unstable sternum, is often left up to the individual surgeon, as the literature is limited to case reports only [7, 8]. Central sternal lucency and loss of sternal wire alignment on post-operative chest X-rays should alert the clinician of sternal malunion/dehiscence. We recommend that sternal wire fractures in this setting should be removed given the potential of angulation and penetration into the mediastinum. In our patient, after repairing the cardiac wound, the sternal bone was repaired using sternal plates.

## Conclusion

In conclusion, recognizing and managing an unstable sternum in patients who have undergone a recent sternotomy is important. A combination of a broken sternal wire with sternal instability may lead to serious and potentially life-threatening complications such as cardiac perforation from an angulated sternal wire. Prompt diagnosis and surgical intervention are essential in treatment in such patients.

### Electronic supplementary material

Below is the link to the electronic supplementary material.

Supplementary Material 1

## Data Availability

Not applicable.

## Abbreviations

CTA: Computed tomography angiography
TEE: Transesophageal echocardiogram
